# Microbial community structure and functional characteristics across the mucosal surfaces of olive flounder (*Paralichthys olivaceus*)

**DOI:** 10.3389/fmicb.2025.1587288

**Published:** 2025-05-21

**Authors:** Jihyun Yu, Min Joo Kang, Mi-Jeong Park, Gyeong Hak Han, Yun Jae Kim, Choong Hwan Noh, Kae Kyoung Kwon

**Affiliations:** ^1^Korea Institute of Ocean Science and Technology, Busan, Republic of Korea; ^2^KIOST School, University of Science and Technology, Daejeon, Republic of Korea

**Keywords:** aquaculture, mucosal surface, 16S rRNA gene, olive flounder, microbiota, functional prediction

## Abstract

The mucosal surfaces of aquatic animals serve as a functional barrier between the host and the aquatic environment, hosting diverse microbial communities that play pivotal roles in host health. In this study, amplicon libraries of the 16S rRNA gene were constructed to investigate the compositional differences between the microbial communities presented in four mucosal surfaces (gills, skin, gut, and ovary) of spawning female olive flounder. To elucidate the unique roles of commensal microbes in external and internal organs, we used PICRUSt2 and ALDEx2, respectively, to predict metabolic functions and identify differential abundances of microbes. Microbial richness was significantly higher in the gills and skin, which are directly exposed to seawater, compared to the intestine and ovary, which are relatively oxygen-poor internal environments. Compositional analysis revealed that the gill mucus was dominated by unclassified *Comamonadaceae*, a member of the *Burkholderiales*. While skin mucus shared constituents with gills and intestines, it also harbored unique taxa, including obligate intracellular parasites such as *Rickettsiales* and *Chlamydiales*. In contrast, the intestinal and ovarian mucus were dominated by the family *Vibrionaceae* of *Gammaproteobacteria*. Functional predictions highlighted the distinct ecological roles of the mucosal microbiota. The gills and skin were enriched in functions related to denitrification, sulfur oxidation, B-group vitamin synthesis, and photosynthesis, reflecting their adaptation to external environmental exposure. Conversely, the intestine was characterized by functions such as beta-lactamase and phenicol resistance, glycosidases, LPS synthesis, and vitamin K2 production. These findings support the idea that while the microbiota of internal organs primarily supports digestive and immunological processes, the symbionts of external organs may play crucial roles in neutralizing toxic compounds from aquaculture practices, such as reduced nitrogen and sulfur compounds, and maintaining the integrity of the mucosal barrier.

## Introduction

The modern aquaculture industry plays a critical role in meeting the escalating demand for seafood while effectively managing limited marine resources. In South Korea, the olive flounder stands out as one of the most popular aquaculture species, holding a substantial portion of the aquaculture production (KOSIS, 2024). In 2022, the olive flounder accounted for 54.9% of South Korea’s total aquaculture production yield, claiming the top among all species in generated product revenue (KOSIS, 2024). This species inhabits coastal areas of Eastern Asia at depths up to 100 meters, primarily feeding on small fish and crustaceans (Bai and Lee, 2010). In South Korea, they are predominantly cultured in land-based tanks along the southern coast, particularly in the South Sea and Jeju Island. Microbiome research in teleost fish has been continuously explored, highlighting its revealing roles in host stress, health, reproduction, and disease resistance (Gioacchini et al., 2010; Roeselers et al., 2011; Boutin et al., 2013; Llewellyn et al., 2014; Perry et al., 2020). Accordingly, efforts to develop probiotics based on microbiome research are actively underway, focusing on specific microbial groups that reduce pathogen infection and mortality rates and promote individual growth (Macey and Coyne, 2005; Vendrell et al., 2008; Klakegg et al., 2020; Ghanei-Motlagh et al., 2021). Developing probiotics and prebiotics is a sustainable solution to reduce dependence on antibiotics and chemical agents in aquaculture.

Skin, gills, and intestines are three representative mucosal surfaces of aquatic animals. These mucosal surfaces are directly exposed to various water quality conditions such as dissolved oxygen, temperature, and salinity. Mucosal surfaces perform vital physiological functions within aquatic animals, such as nutrient absorption, osmoregulation, and waste disposal (Eric Peatman, 2015). One of their most important roles is to serve as primary barriers against pathogen invasion (Shephard, 1994; Dalmo et al., 1997; Ellis, 2001). The symbiotic microorganisms inhabiting mucosal surfaces can protect the host by directly inhibiting the growth of pathogenic bacteria or by competing for physical resources in the ecological niche (Jöborn et al., 1997). Moreover, the mucosal surfaces of aquatic organisms can serve as a pathway for introducing immunomodulators, probiotics/prebiotics, or mucosal vaccines through immersion/deep methods (Eric Peatman, 2015; Esteban Soto and Tobar, 2015). The composition of mucosal microbiota in the gills, skin, and intestines in some of the world’s most popular aquaculture species, including yellowtail, Atlantic salmon, European seabass, and gilthead seabream has been investigated (Legrand et al., 2018; Rosado et al., 2019a; Minich et al., 2020; Rosado et al., 2021; Bledsoe et al., 2022; Rosado et al., 2023). Previous studies have shown that factors such as stress, pathogens, or antibiotic administration induce distinct responses at mucosal sites, leading to significant changes in microbial composition and diversity (Boutin et al., 2013; Rosado et al., 2019b; Rosado et al., 2023). While the role and function of mucosal surfaces have a significant impact on the health of aquatic animals compared to terrestrial animals, the majority of the research has been focused on the intestinal microbiota. Recently, studies on the microbiome of olive flounder have been explored under various aspects and conditions. Key investigations include characteristics during early life stages, comparisons between wild and farmed specimens, and the influence of feed composition (Kim and Kim, 2013; Jiang et al., 2019; Niu et al., 2020; Jang et al., 2022). Additionally, there have been continuous attempts to apply probiotic candidates. Probiotics tested in olive flounder include *Bacillus* sp., *Lactobacillus* sp., *Rummeliibacillus* sp., *Microbacterium* sp., and a combination of *Gluconacetobacter* sp. and the budding yeasts *Groenewaldozyma salmanticensis*. These probiotics have been evaluated for their effects on disease resistance, feed utilization efficiency, and immunostimulatory activity (Kim et al., 2013; Jang et al., 2019; Jang et al., 2020; Rhee et al., 2020; Jang et al., 2021; Niu et al., 2021; Lee et al., 2023; Gao et al., 2024). Despite increasing studies on the microbiome of olive flounder, research has still primarily focused on the intestine. Considering the importance of mucosal surfaces in aquatic organisms, exploring the microbiome of mucosal sites in the olive flounder is essential. This study aimed to identify the microbial composition and predict the function of microbial communities inhabiting four mucosal sites (gill, skin, intestine, and ovary) of the olive flounder, the major aquaculture species in Korea. The results of this study provide insights that will elevate our understanding of the microbiome of mucosal surfaces in aquatic animals and aid in the development of environmentally friendly aquaculture technologies.

## Materials and methods

### Sample collection

We collected olive flounders raised at a land-based commercial aquaculture facility (Haeyeon Fisheries) in Jeju Island, South Korea. The olive flounders were 1–2 years old, spawning females, and some were undergoing gonadal degradation and absorption processes. Experimental fish were sampled on the same day, ten individuals from each culture tank (tanks C and L). Both tanks were supplied with the same commercial feed and seawater, and the fish in both tanks were derived from the same parental stock and raised at the same developmental stage. Fish were euthanized with 600 ppm Tricaine-S (Syndel Laboratories Ltd., Ferndale, WA, USA). Blood samples were collected from the caudal vein using a sterile 3-ml volume syringe coated with 0.5 M ethylene diamine tetraacetic acid (EDTA, pH 8.0; Bioneer, Daejeon, Korea) anticoagulant. Plasma samples were obtained by centrifugation at 18,213 rcf for 10 min at 4°C (Centrifuge 5,427 R, Eppendorf, Hamburg, Germany) and stored at −78°C in a deep freezer (NF-140SF, Nihon Freezer Co., Ltd., Japan) until analysis. For microbiome analysis, we obtained mucus samples from the gills, skin, intestines, and ovaries of individual fish. The gills and skin of the olive flounder were rinsed with sterile PBS to remove seawater before the sampling process. Gill mucus was collected by gently swabbing the second and third gill arches several times with a sterile swab, and the swab tip was then stored in sterile PBS. To collect skin mucus, the entire upper surface of the fish was gently scraped in the direction of the scales using a sterile cell lifter (Costar® 3,008, 18 cm, Corning® International, Mexico), and the collected mucus was stored in sterile PBS. Ovarian mucus was collected by inserting a sterilized swab into the ovary and gently swabbing it multiple times. For intestinal mucus collection, the olive flounders were dissected using sterilized dissecting instruments. A 4–5 cm in long segment of the intestine from the anus was separated from the entire intestine, and the intestinal contents were scraped using sterilized forceps and stored in sterile PBS. All mucus samples were transported below −20°C and stored at −80°C until DNA extraction. Additionally, for environmental association analysis, commercial feed used in aquaculture, inflowing seawater to the aquaculture facility, and seawater from each rearing tank were collected. All seawater samples were filtered through a stainless wire mesh net with a pore size of 63 μm, followed by filtration through a 0.2 μm Poly Carbonate Track Etched (PCTE) membrane disk (diameter 47 mm, GVS, USA), and the PCTE filters were stored at −80°C until DNA extraction.

### Blood biochemical analysis

Plasma glucose (mg/dL), glutamic oxaloacetic transaminase (GOT, U/I), and glutamic pyruvate transaminase (GPT, U/I) concentrations were determined in duplicate using an automatic analyzer (FUJI DRI-CHEM 4000i, Fujifilm Co., Tokyo, Japan). The automatic analyzer was operated according to the manufacturer’s protocol using selected testing slides (GLU-PIII, GOT/AST-PIII, and GPT/ALT-PIII, Fujifilm Co., Tokyo, Japan). Plasma cortisol and estradiol-17β concentration were measured in duplicate by enzyme-linked immunosorbent assay (ELISA) using the fish cortisol ELISA kit (CSB-E08487f, Cusabio Technology, Houston, USA) and the fish estradiol (E2) ELISA kit (CSB-E13017Fh, Cusabio Technology, Houston, USA), respectively. Absorbance was measured at 450 nm using a microplate reader (Synergy HTX multi-mode reader with Gen5 software, Biotek, Winooski, USA).

### DNA extraction and amplicon sequencing

Mucus samples stored at −80°C were thawed and homogenized using a vortex mixer. Then, the swab tips were removed from the mucus, and total DNA was extracted using the QIAamp® Fast DNA Stool Mini Kit (QIAGEN, Germany). Total DNA from PCTE filters and commercial feed were extracted using DNeasy PowerSoil Pro Kits (QIAGEN, Germany). Polymerase chain reaction (PCR) targeting the V3-V4 region of the 16S rRNA gene was then performed using DNA extracted from individual samples as a template. The universal primer set for PCR amplification comprised 341F (5’-CCTACGGGNBGCASCAG-3′) and 805R (5’-GACTACNVGGGTATCTAAT-3′) (Takahashi et al., 2014). PCR products were purified using the QIAquick® PCR Purification Kit (QIAGEN, Germany) and quantified using the Qubit™ 4 fluorometer (Invitrogen, USA). Libraries were constructed from the samples meeting the concentration criteria. Paired-end (300 bp) sequencing was commercially conducted at the NICEM (Seoul, Korea) using the Illumina MiSeq platform.

### Data processing and general analysis

The obtained raw sequences were prepared as FASTQ files following the cassava 1.8 format and then demultiplexed and merged according to the Qiime2 pipeline (Bolyen et al., 2019). At first, primer sequences were trimmed using Cutadapt, and chimeric sequences were removed using DADA2 and VSEARCH (Martin, 2011; Callahan et al., 2016; Rognes et al., 2016). Taxonomic assignment of discovered Amplicon Sequence Variants (ASVs) was conducted using the SILVA database (Silva-138-99-classifier) (Quast et al., 2012). Subsequent precise taxonomic classification was referenced from the List of Prokaryotic names with Standing in Nomenclature (LPSN) (Parte et al., 2020). Alpha diversity indices were calculated using the phyloseq R package (v.1.16.2), visualized using ggplot2, and differences between groups were tested using the Wilcoxon Rank-Sum test (McMurdie and Holmes, 2013; Wickham, 2016). MEGA11 was employed to construct trees using the Unweighted Pair Group Method with Arithmetic Mean (UPGMA) method based on the Jaccard distance matrix obtained from beta diversity statistics of Qiime2 results (Tamura et al., 2021). Alluvial plots describing the relative abundance of taxa within each group were generated using the ggalluvial R package (v.0.12.4) (Brunson, 2020). Complex heatmap analysis was performed using the microViz R package (v.0.12.1), where read count data were log2 transformed, and the distance metrics used was calculated by the default Euclidean method (Barnett et al., 2021). Principal coordinates analysis (PCoA) using the microViz R package (v.0.12.1) was conducted, with calculations based on Aitchison distances introducing count data (Barnett et al., 2021). Network analysis was conducted using the NetCoMi R package (v.1.1.0), based on a Pearson correlation of center log-ratio (CLR) transformed data (Peschel et al., 2021). Multivariable association analysis between microbial composition and physiological markers, including morphometric and blood biochemical variables, was conducted using MaAsLin2 R package (v.1.7.3). For analysis, we applied the Cumulative Sum Scaling (CSS) normalization method and utilized a Negative Binomial Model (NEGBIN) for count data with a minimum abundance threshold of 100 (Mallick et al., 2021). To investigate the environmental contributions to the microbial communities of olive flounder, we applied the R package FEAST (v.0.1.0), a probabilistic source tracking tool based on an expectation–maximization algorithm (Shenhav et al., 2019). Functional prediction of microbial communities was performed by introducing PICRUSt2 (Douglas et al., 2020). The predicted microbial metabolites and functions were analyzed by referring to the Kyoto Encyclopedia of Genes and Genomes (KEGG) orthologues and modules (Kanehisa et al., 2016). Specifically, short-chain fatty acid (SCFA) related KEGG orthologs (KOs) were referred from Zhang et al., 2021, while KOs related to vitamin K and B group vitamins were cited from Jiang et al., 2022. Differential abundance (DA) analysis of taxa composition and PICRUSt2 results between groups was conducted using ALDEx2 v.1.34.0 (Fernandes et al., 2014) on untransformed read count data. *p*-values were adjusted using the Benjamini-Hochberg (BH) method. Statistical comparisons in the DA analysis were performed using the Wilcoxon rank-sum test for two-group comparisons and the Kruskal-Wallis test was chosen for three groups.

## Results

### Data summary

Olive flounders from two rearing tanks (C and L) had average weights and lengths of 1,364.70 ± 73.82 g and 48.40 ± 1.15 cm, respectively (expressed as mean and standard error). Experimental fish sampled from the two tanks had no statistically significant differences in weight, length, blood glucose levels, or liver indices (Supplementary Table 1). For the analysis of the microbial community, a total of 51 samples meeting quality standards were sequenced, including mucus collected from four different body sites of olive flounders (total 47 samples: 11 from gills, 18 from skin, 13 from intestine, and five from ovaries) as well as three seawater (1 from inflow seawater, and two from rearing tanks) and from the commercial feed. After the removal of low-quality reads and unassigned sequences, a total of 5,213,830 high-quality reads (ranging from 18,721 to 190,705 reads) were obtained from the entire sequencing dataset (*n* = 51), which were assigned to a total of 7,922 ASVs. As for the detailed sequencing results, refer to Supplementary Table 2. Rarefaction curve analysis confirmed that the sequencing depth was sufficient to capture the microbial diversity across all sample types (Supplementary Figure 1). Among these, eight individual fish (R8 group: C2, 5, 6 and L1, 2, 3, 6, 10) each providing mucosal samples from the gills, skin, and intestine (*n* = 24) were selected to compare the microbial composition and functional characteristics across different body sites, irrespective of individual variation of fish.

### Diversity analysis results

**Figure 1:**
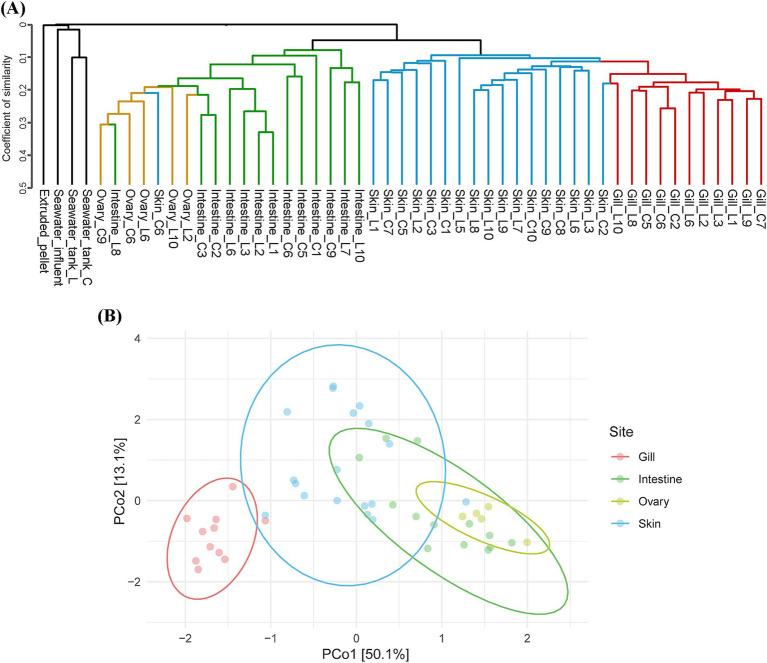
Beta diversity summary. **(A)** Dendrogram of all microbial communities across environmental factors and olive flounder, based on Jaccard distance. **(B)** Principal coordinate analysis (PCoA) based on Aitchison distance, illustrating the microbial communities of four different mucosal surfaces. Each point represents an individual sample, and each color corresponds to a specific site.

Beta diversity analysis based on the Jaccard distance tree revealed distinct clustering patterns and hierarchical relationships among the total microbial community samples (see Figure 1A). Among environmental samples, seawater samples from two rearing tanks clustered together, distinct from inflow seawater, and also formed a separate branch from the feed as well. Samples from olive flounders showed distinct branching by mucosal sites, with clusters primarily comprising gills and skin and a separate cluster comprising intestines and ovaries. While mucus samples from the same body sites tended to cluster closely together, an exception was observed where the skin microbiota of C6 clustered with the intestine/ovary cluster (Figure 1A). The NMDS analysis provides an intuitive visualization of the distribution patterns and relationships between the different mucosal sites (Figure 1B). The clustering pattern for gill samples showed low intra-group variation. In contrast, the skin samples exhibited a more comprehensive range of variation. Moreover, the NMDS analysis showed that the skin cluster closely resembles the intestinal cluster, highlighting a less evident relationship in the Jaccard distance tree (Figure 1B).

The alpha diversity of overall samples was assessed using the Chao1 and Shannon indices to represent microbial richness and diversity, respectively (Supplementary Figure 2). Microbial communities isolated from olive flounders exhibited a trend of relatively higher species richness in gills and skin and lower richness in intestine and ovaries (Supplementary Figure 2). Significant differences in alpha diversity among the three body sites (gills, skin, intestine) were verified using the Wilcoxon rank sum test in the R8 group. The gill microbiota displayed the highest microbial richness, with both the gills and skin exhibiting significantly higher richness than the intestinal microbial community. However, regarding diversity, the gill microbiota showed the lowest level and showed a significant difference compared to the skin, with a confidence level exceeding 95%. The microbial diversity of seawater samples was exceptionally high compared to olive flounder and feed. However, microbial richness and diversity decreased sharply after entering the rearing tanks (C, L) (Supplementary Figure 2).

### Microbial community structure across body sites of olive flounder

**Figure 2:**
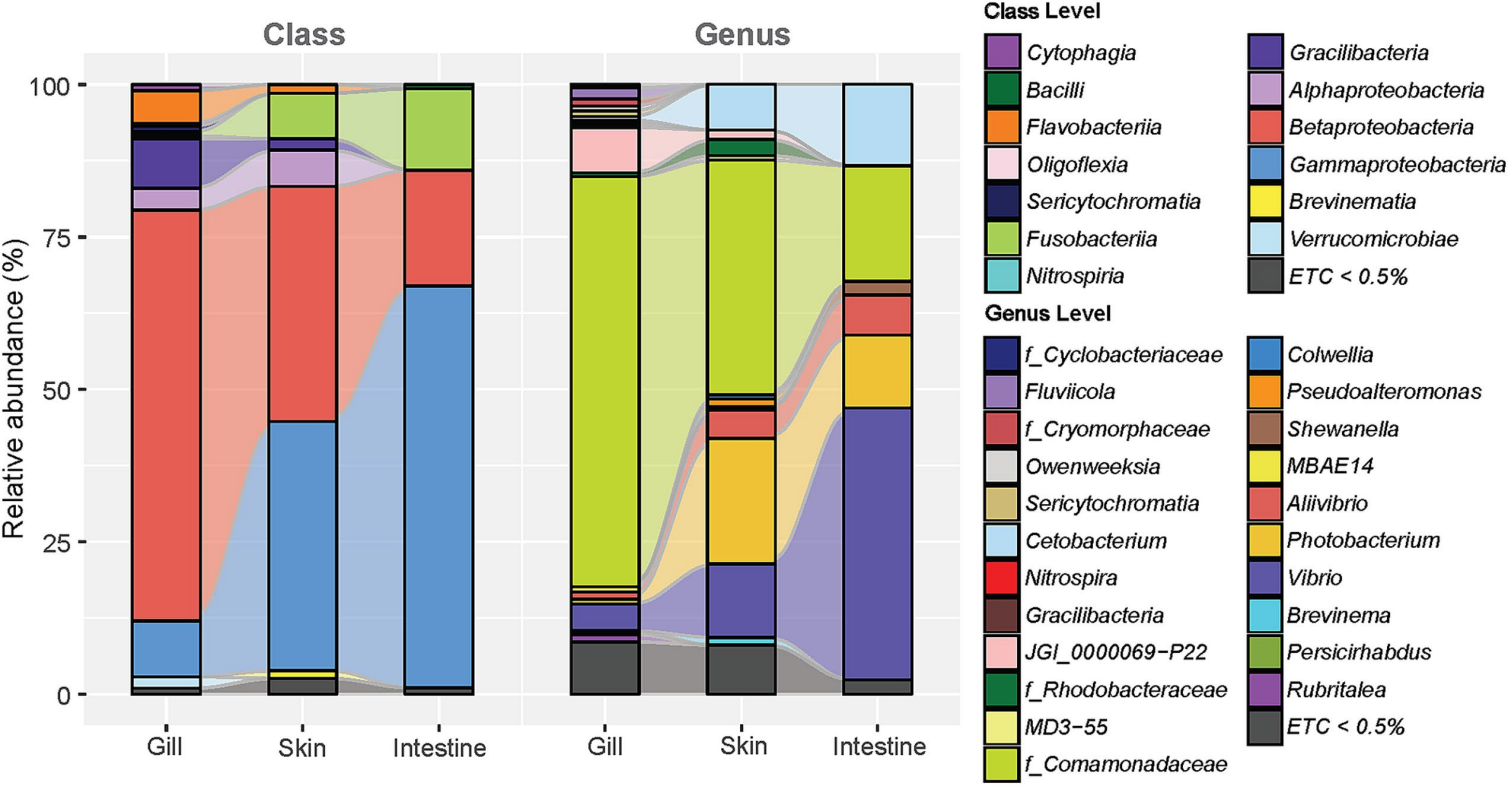
Alluvial plot illustrating the average relative abundance of microbial composition at three different sites in R8 group olive flounder (comprising eight fish: C2, 5, 6, and L1, 2, 3, 6, 10). The plot includes classifications at the class and genus levels for taxa constituting more than 0.5% of the average relative abundance at each site.

We analyzed the microbial composition of four mucosal sites of the olive flounder (gills, skin, intestines, and ovaries), as well as their feed, rearing seawater, and the seawater entering the aquaculture system (Supplementary Figure 3). Majority of microbial communities found in the olive flounder were predominantly composed of *Pseudomonadota*, with relative abundances of group mean with standard deviation as follows: gills 79.23 ± 8.15% (*n* = 11), skin 85.09 ± 7.35% (*n* = 18), intestines 86.85 ± 17.43% (*n* = 13), and ovaries 87.56 ± 14.91% (*n* = 5). At the class level, *Pseudomonadota* was mostly comprised of *Gammaproteobacteria* and *Betaproteobacteria*. *Betaproteobacteria* were most prevalent in the gills (69.39 ± 10.79%), whereas *Gammaproteobacteria* were most dominant in the ovaries (86.54 ± 14.97%). The microbial composition of different mucosal sites of olive flounder exhibited an increase or decrease in the abundance of shared taxa, in the order of gills, skin, intestines, and ovaries. To illustrate this tendency without individual variation of fish, we selected the microbial composition of the gill, skin, and intestinal mucus from the R8 group of olive flounder and displayed it in an alluvial plot (Figure 2). The microbial composition of the gills in the R8 group of olive flounder exhibited the most diverse class variation among the three mucosal sites (Figure 2). Apart from the most prevalent component across all three sites, *Gammaproteobacteria* and *Betaproteobacteria*, the gills shared *Gracilibacteria*, *Flavobacteriia*, and *Alphaproteobacteria* with the skin (Figure 2). In contrast, the intestines displayed a much simpler composition, with *Gammaproteobacteria*, *Betaproteobacteria*, and *Fusobacteria* collectively accounting for 98.30% (*n* = 8) of the total reads (Figure 2). The skin also shared considerable amounts of *Fusobacteria* with the intestines, exhibited intermediate characteristics between the gills and intestines (Figure 2). At the genus level, the most abundant component of the microbiom of olive flounder was an unclassified member of the family *Comamonadaceae*. This accounted for most of the reads assigned to *Betaproteobacteria* at the class level and *Burkholderiales* at the order level. Unclassified *Burkholderiales* have been reported as a member of the microbial communities in the skin and gills of seabream and seabass, while unclassified *Comamonadaceae* were prevalent in the skin and intestines of gilthead seabream (Rosado et al., 2021; Thomas et al., 2023). *Comamonadaceae*_uc was consistently found in all parts of the olive flounder, comprising 67.30 ± 11.15% in the gills, 38.50 ± 23.71% in the skin, and 18.96 ± 26.82% in the intestines of the R8 group. Following *Comamonadaceae*_uc, the second most abundant genus in the olive flounder was *Vibrio*, which is known to be a general constituent of the intestinal tract of marine animals and the marine environment. This genus accounted for 44.59 ± 32.14% of the intestinal microbiota in the R8 group, with lower proportions in the skin (12.05 ± 7.94%) and gills (4.36 ± 7.08%). Other members of *Gammaproteobacteria*, such as *Photobacterium* and *Aliivibrio*, were prevalently found in the intestines and skin but rarely in the gills. Similarly, both skin and intestines shared a considerable amount of *Cetobacterium*, which comprises most of the reads assigned to the *Fusobacteria* class. Genus *Cetobacterium* was most prevalent in the intestines (13.37 ± 18.39%), followed by skin (7.50 ± 11.39%), and least abundant in the gills (0.6 ± 1.27%). In contrast, JGI_0000069_P22 of the class *Gracilibacteria* was most frequently found in the gills (7.45 ± 1.91%), with minimal presence in the skin (1.53 ± 1.85%) and nearly absent in the intestines (0.05 ± 0.13%). The skin tended to encompass most of the taxa present in both the gills and intestines. However, it was distinguished by specific taxa at the genus level, such as *Rdobacteraceae*_uc (1.00 ± 1.57%), *Pseudoalteromonas* (1.29 ± 2.36%), and *Brevinema* (1.25 ± 2.00%).

### Heatmap analysis

**Figure 3:**
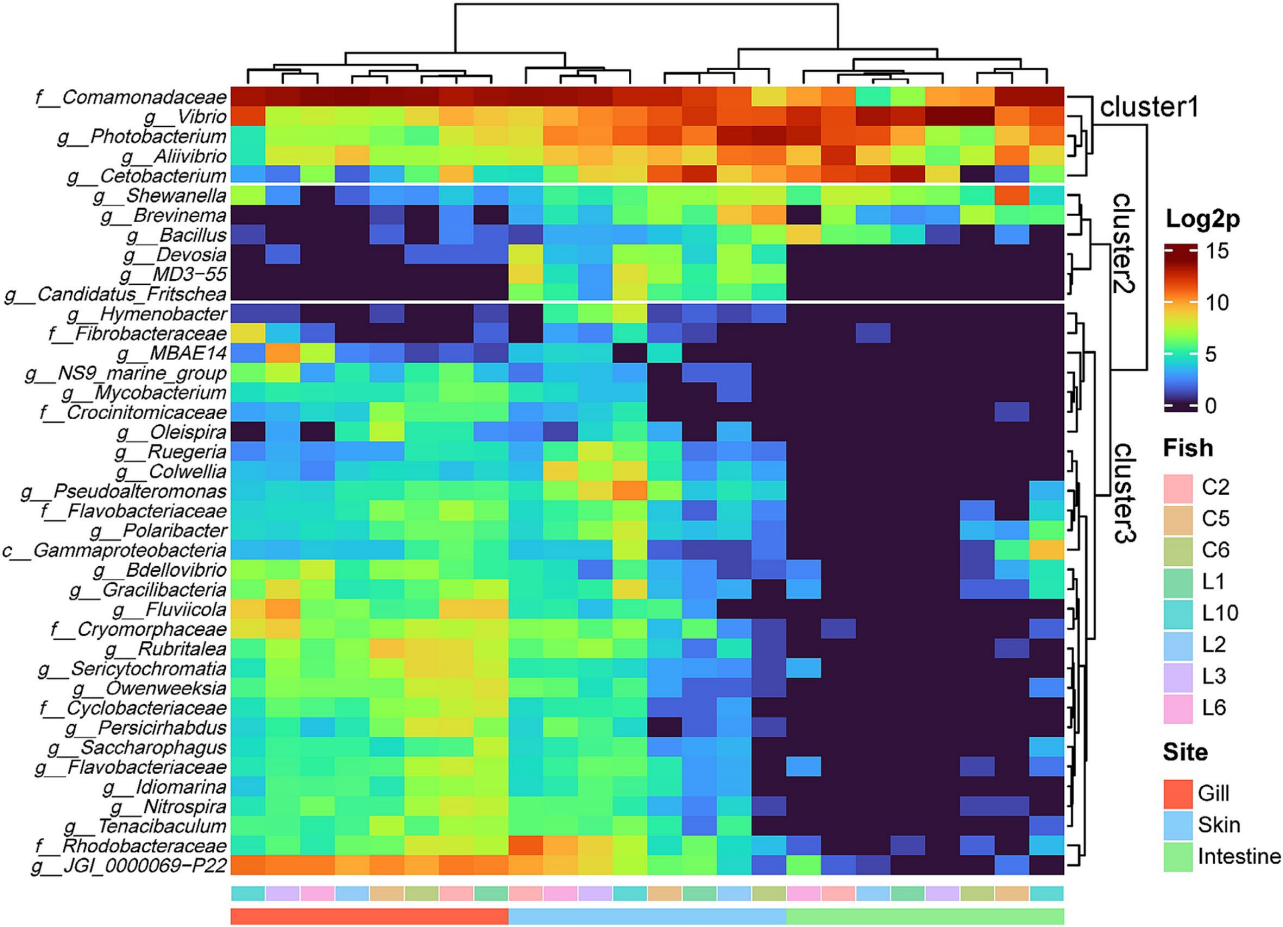
Complex heatmap of microbial composition for the top 40 genera in three sites of the R8 group olive flounder. Count data were log2 transformed and analyzed based on Euclidean distance.

The complex heatmap analysis illustrated the clustering pattern based on existence of most prevalent taxa at the genus level of individual samples within the R8 group (Figure 3). First, it showed that samples from three sites were branched into two main clusters, represented by the intestine and gills. Skin samples were evenly distributed in two clusters. On the other hand, three major clusters were identified within the taxa at the genus level. The first cluster comprised the five most prevalent genera, including *Comamonadaceae*_uc, *Vibrio*, *Photobacterium*, *Aliivibrio*, and *Cetobacterium*, which were consistently detected across all samples (Figure 3). The second cluster consisted of six genera commonly found in skin samples. Cluster 2 includes *Shewanella*, *Brevinema*, *Bacillus*, *Devosia*, MD3-55, and *Candidatus* Fritschea. Among them, MD3-55 and *Candidatus* Fritschea are exclusively found in the skin samples. Cluster 3, represented by JGI_0000069 − P22, identified the distinctive composition of genera predominantly found in gills and a portion of skins but rarely in intestine (Figure 3). It encompassed the most diverse range of the phyla composition, comprising 11 *Bacteroidota*, 9 *Pseudomonadota*, two each of *Verrucomicrobiota* and *Patescibacteria*, and one each of *Actinobacteriota*, *Bdellovibrionota*, *Cyanobacteria*, *Nitrospirota*, and *Fibrobacterota* (Figure 3). As mentioned earlier, unlike gills and intestines form independent branches, skin samples were distributed in the gill and intestinal clusters, with samples corresponding to individuals C2, L6, L3, and L10 being included in the gill cluster and samples corresponding to individuals C5, L1, L2, and C6 being included in the intestinal cluster (Figure 3). These divaricate skin samples showed distinct patterns in the distribution of specific taxa. Skin samples clustering with the gills exhibited a higher presence of Cluster 3 constituents, especially JGI 000069-P22 and *Rhodobacteraceae*. Conversely, samples clustering with intestines showed a lower presence overall in Cluster 3, with a relative prominence of *Cetobacterium, Photobacterium*, and *Brevinema* from Clusters 1 and 2 (Figure 3). There was no correlation among the same fish individuals observed in the clustering pattern.

### Predicted functional differences across three mucosal sites of olive flounder

**Figure 4:**
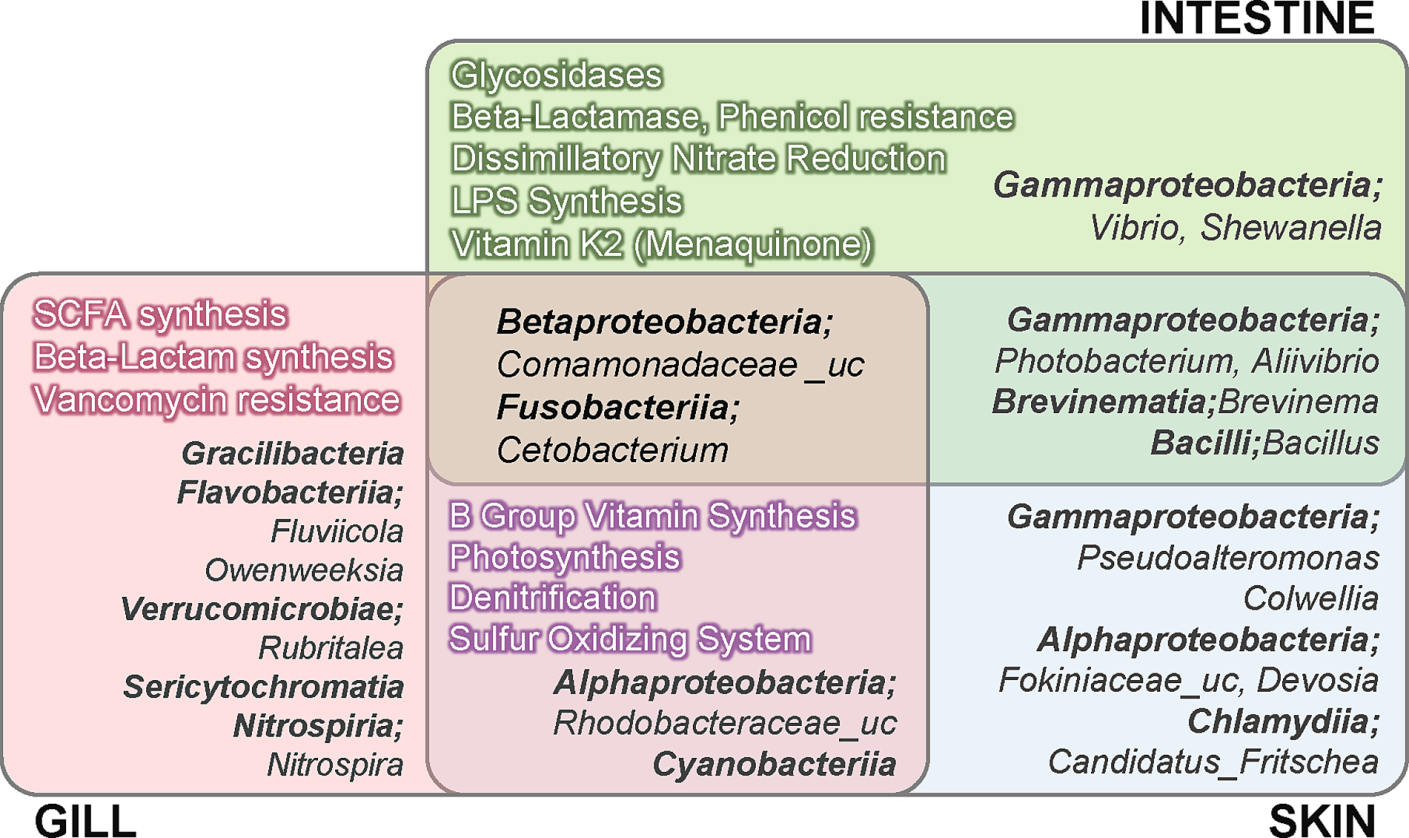
Summary of the functional and compositional characteristics of the microbiota across three different mucosal sites in R8 group olive flounder, based on differential abundance (DA) analysis. The DA analysis was conducted using the ALDEx2 tool with read count data from amplicon sequencing and predicted functional profiles. Taxa and functions displayed in the figure were filtered according to abundance and statistical significance criteria, as described in Supplementary Table 5.

Based on the analysis of microbial communities, we performed functional predictions using PICRUSt2. The analysis identified 7,831 KOs across olive flounder and environmental specimens (Supplementary Table 3). Accordingly, we examined whether there were significant site-specific differences in the mucosal surface regarding the ability to synthesize host-beneficial metabolites, such as vitamins, SCFAs, antibiotics, and digestive enzymes, as well as in microbial energy metabolism, including nitrogen and sulfur cycling and photosynthesis, and in the synthesis and resistance of various antibiotics, by referring to KEGG Orthologs (KOs) based on functional prediction results. The detailed table of KOs corresponding to each function utilized in the analysis is provided in Supplementary Table 4. The analysis was conducted on groups of KOs present in at least one of the three mucosal surfaces (gills, skin, intestine) of R8 group olive flounder. The differential abundance of functional features was assessed using the ALDEx2 tool, and statistically significant differences (adjusted *p* < 0.05) were determined using the Kruskal-Wallis and Wilcoxon rank-sum tests. Only functions meeting these criteria were visualized in Figure 4, and the complete list of tested features is provided in Supplementary Table 5. Among the three sites, the intestine exhibited a significantly higher abundance of glycosidases (EC 3.2.1.-) related to carbohydrates and organic carbon decomposition, dissimilatory nitrate reduction, lipopolysaccharide (LPS) synthesis, and vitamin K2 (menaquinone) production (Figure 4). In contrast, the external organs (skin and gills) were associated with photosynthesis, denitrification, sulfur oxidation, and the overall synthesis of B-group vitamins compared to the intestine. The gills shared most functional characteristics with the skin but exhibited significantly higher levels of SCFA synthetic enzymes and *β*-lactam antibiotic synthetic enzymes among the three sites (Figure 4). KOs related to antibiotic resistance and biosynthesis exhibited distinct differences across mucosal sites. Regarding antibiotic resistance, the predicted abundance of resistance genes for aminoglycosides, β-lactam antibiotics, phenicols, tetracyclines, trimethoprim, and vancomycin varied significantly among sites. Among these, resistance genes against phenicols and β-lactam antibiotics were particularly abundant in the intestine, whereas vancomycin resistance genes were most abundant in the gills. In terms of KOs associated with antibiotic biosynthesis, genes involved in fosfomycin and monobactam (nocardicin A) biosynthesis were significantly enriched in the gills, while bacilysin biosynthesis genes were more abundant in the intestine and skin compared to the gills. Distinct patterns were also observed for genes involved in nitrogen and sulfur cycling across mucosal sites. In the intestine, genes associated with dissimilatory nitrate reduction (e.g., nitrate reductase *napAB* and *nrfA*) were predicted to be significantly more abundant compared to the other two mucosal sites. Conversely, the external organs (gills and skin) exhibited significantly higher levels of genes associated with the denitrification pathway compared to the intestine. Furthermore, among the three sites, the gills displayed the highest abundance of *nirK*, a core enzyme in the denitrification pathway. Regarding sulfur metabolism, genes involved in the assimilatory sulfate reduction pathway were commonly distributed across all mucosal sites. However, significant differences were observed in the types and abundance of these genes between external and internal organs. Among the three sites, the gills exhibited the highest predicted abundance of *sir* (sulfite reductase [ferredoxin]), while the intestine showed the highest levels of *cysJ* (sulfite reductase [NADPH]) and *cysC* (adenylylsulfate kinase). Furthermore, genes related to the sulfur oxidation system were predicted to be significantly more abundant in the external organs compared to the intestine.

### Differential microbial abundance across three mucosal sites of olive flounder

The differential abundance analysis of microbial composition across the three mucosal surfaces of the R8 group olive flounder revealed distinct characteristics (Figure 4). The intestine exhibited a significantly higher abundance of the genera *Vibrio* and *Shewanella*, both belonging to the class *Gammaproteobacteria*. The skin shared a substantial number of taxa with the intestine, including *Photobacterium* and *Aliivibrio* from *Gammaproteobacteria*, as well as *Brevinema* and *Bacillus*. Distinctive taxa identified on the skin included *Pseudoalteromonas* and *Colwellia* from *Gammaproteobacteria*, *Fokiniaceae*_uc, and *Devosia* from *Alphaproteobacteria* and *Candidatus* Fritschea from *Chlamydiia*. In contrast, the gills harbored the most diverse range of unique microbial taxa, including members of the classes *Gracilibacteria*, *Flavobacteriia*, *Verrucomicrobiae*, and *Sericytochromatia*. The skin and gills exhibited a distinct microbial composition compared to the intestine, notably including members of *Cyanobacteria* and *Rhodobacteraceae*. Core taxa consistently present across all three sites of the olive flounder, without significant differences in abundance, were *Comamonadaceae*_uc and *Cetobacterium*.

### Network analysis on the microbial community of olive flounder

**Figure 5:**
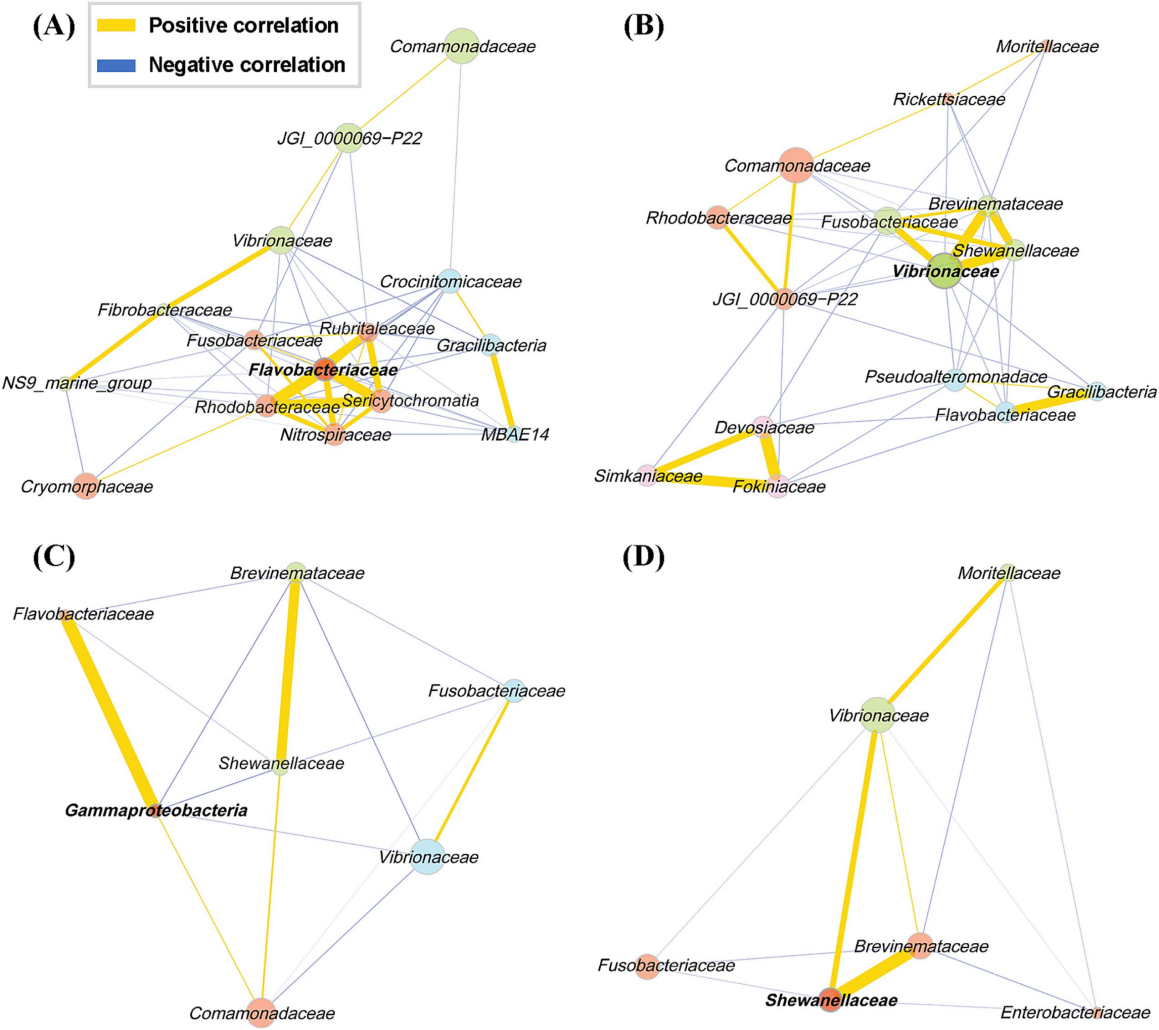
Pearson correlation network of the families present in each of the four mucosal surfaces of the olive flounder. Networks include only associations with an absolute Pearson correlation coefficient of 0.3 or higher. Each node represents a bacterial family, and node colors indicate clusters of co-occurring taxa identified through community detection. Bold-labeled nodes represent cluster hubs, which are key taxa with high centrality within each community. Edge colors denote the type of correlation: yellow for positive and blue for negative, as shown in the legend. Edge thickness reflects the strength of the correlation. **(A)** Gill, **(B)** skin, **(C)** intestine, **(D)** ovary.

We performed a network analysis based on the correlation among microbial taxa identified in different body sites of the olive flounder (Figure 5). The Network analysis was conducted using the top 15 families in terms of abundance at each site, calculated via the Pearson correlation method. Only associations with a correlation coefficient of 0.3 or higher were included. The network analysis allows us to infer ecological interactions, particularly potential cooperative or competitive relationships between microbial taxa across different mucosal sites of olive flounder. The microbial co-occurrence network in each site of the olive flounder was composed of 2 to 4 clusters, with a single hub for each site (Figure 5). The hub family for each site displayed unique characteristics: *Flavobacteriaceae* in the gills, *Vibrionaceae* on the skin, an unclassified member of *Gammaproteobacteria* in the intestines, and *Shewanellaceae* in the ovaries (Figure 5). The central cluster in the gills included six Families (*Flavobacteriaceae*, *Rhodobacteraceae*, *Sericytochromatia*, *Nitrospiraceae*, *Rubritaleaceae*, and *Fusobacteriaceae*) that were all strongly positively correlated and originated from different phyla (Figure 5). This cluster was negatively correlated with the other two clusters. Notably, the three predominant taxa in the gill microbiota in order of *Comamonadaceae*, JGI_0000069-P22, and *Vibrionaceae* are included in the same cluster, exhibiting positive correlations (Figure 5). While the skin microbial community was organized into four clusters, with *Vibrionaceae*, the hub family, forming a close positive correlation with *Shewanellaceae*, *Brevinemataceae*, and *Fusobacteriaceae*. Similar to the gills, *Comamonadaceae* in the skin showed positive correlations with JGI_0000069-P22 but a negative correlation with *Vibrionaceae* (Figure 5). Interestingly, *Fokiniaceae* did not correlate significantly with *Rickettsiaceae* from the same order, whereas they were positively correlated with *Simkaniaceae* from *Chlamydiales* and *Devosiaceae* from *Rhizobiales*, forming a distinct cluster (Figure 5). The family composition in the internal organs, such as the intestines and ovaries, was more straightforward. Three clusters were identified in the intestines, with members of the dominant class *Gammaproteobacteria* distributed across the other clusters (Figure 5). The hub taxon, *Gammaproteobacteria*_uc, exhibited positive correlations with *Flavobacteriaceae* and *Comamonadaceae* and negative correlations with *Brevinemataceae*, *Vibrionaceae*, and *Shewanellaceae* (Figure 5). In the ovaries, two clusters were identified, with *Shewanellaceae* as the hub family (Figure 5). This family was present in all sites except the gills and consistently showed a strong positive correlation with *Brevinemataceae*, regardless of the site (Figure 5).

### Microbial association with physiological markers

**Figure 6:**
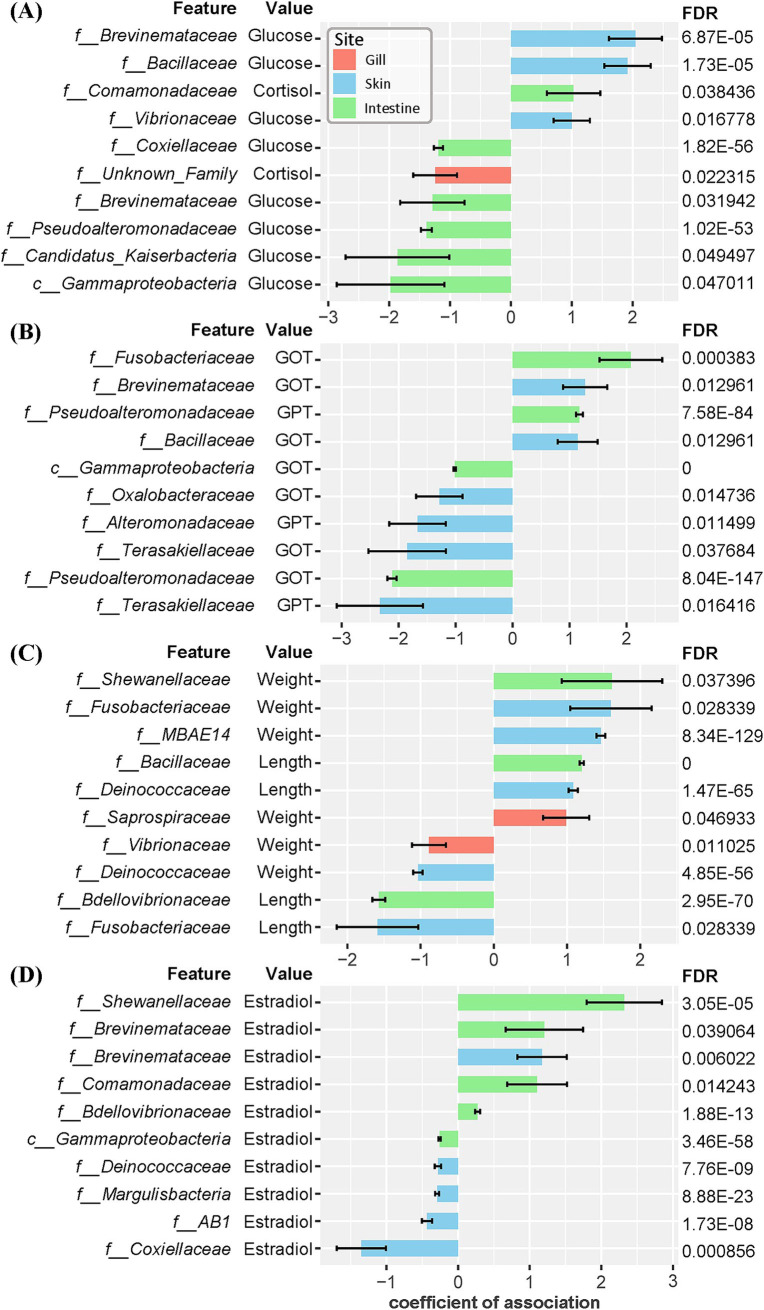
Coefficients of association between microbial composition at the family level across three mucosal sites and morphometric and blood biochemical variables in individual olive flounder. The False Discovery Rate (FDR) was controlled using the Benjamini-Hochberg (BH) method. The error bars represent the standard error of the coefficients. The top 10 associations are described for each group based on the absolute value of coefficients that meet the FDR cutoff of 0.05. **(A)** Associations with stress indices (glucose, cortisol). **(B)** Associations with liver indices (GOT, GPT). **(C)** Associations with morphometric parameters (total length, weight). **(D)** Associations with estrogenic hormone (estradiol-17β).

We conducted a multivariable association analysis to explore the relationships between microbial communities and physiological markers in olive flounder. This analysis focused on the associations between the abundance of microbial families and various physiological markers, including morphometric parameters (body weight and length), stress indicators (cortisol and glucose), liver indices (GOT and GPT), and estrogenic hormone (estradiol-17β). As a result, 119 significant associations were identified across three mucosal sites (gill, skin, intestine) based on the False Discovery Rate (FDR) cutoff of 0.1 (Supplementary Table 6). The skin exhibited the highest number of significant associations (62), followed by the intestine (46), and the gill (11) (Supplementary Table 6). Regarding stress markers, an abundance of *Brevinemataceae* in the skin had the strongest positive association with blood glucose levels (Figure 6). Other families in the skin, such as *Bacillaceae* and *Vibrionaceae*, also showed positive correlations with elevated glucose level. In the intestine, *Comamonadaceae* exhibited a positive correlation with cortisol level, while unclassified *Gammaproteobacteria*, *Candidatus_Kaiserbacteria*, *Pseudoalteromonadaceae*, and *Brevinemataceae* were negatively correlated with glucose level (Figure 6). For liver indices, *Fusobacteriaceae* and *Pseudoalteromonadaceae* in the intestine and *Brevinemataceae* and *Bacillaceae* in the skin showed the strongest positive correlations with increased GOT and GPT levels (Figure 6). Conversely, *Terasakiellaceae*, *Alteromonadaceae*, and *Oxalobacteraceae* in the skin and *Pseudoalteromonadaceae* and Gammaproteobacteria in the intestine exhibited the strongest negative associations with GOT & GPT levels. Notably, *Terasakiellaceae* in the skin was negatively correlated with both GOT and GPT levels (Figure 6). Regarding morphometric parameters such as body weight and length, *Shewanellaceae* and *Bacillaceae* in the intestine, *Fusobacteriaceae*, *MBAE14*, and *Deinococcaceae* in the skin, and *Saprospiraceae* in the gill were most positively associated (Figure 6). Paradoxically, *Fusobacteriaceae* in the skin showed a positive correlation with weight but a negative correlation with length. Finally, associations between microbial communities and the estrogenic hormone estradiol were observed only in the intestine and skin of olive flounder. Families such as *Shewanellaceae*, *Comamonadaceae*, *Brevinemataceae*, and *Bdellovibrionaceae* in the intestine displayed the strongest positive correlations with estradiol levels (Figure 6). The *Brevinemataceae* family was particularly notable, showing a significant association with estradiol in both the intestine and the skin.

### Microbial associations with environmental factors

**Figure 7:**
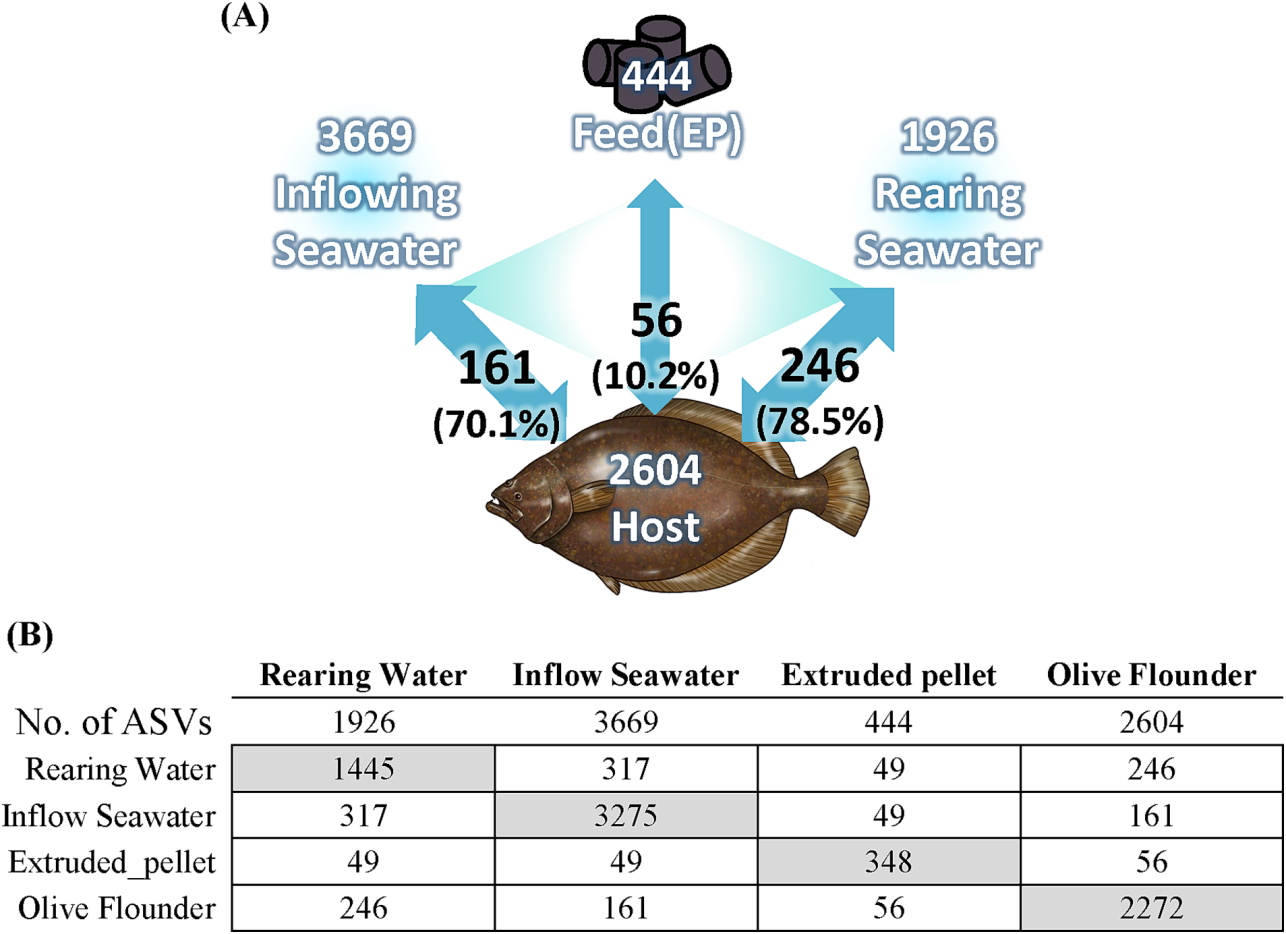
Relationship between olive flounder and environmental factors. **(A)** Diagram illustrating the relationship of olive flounder with seawater samples and feed. White text indicates the number of ASVs present in each group, while black text shows the number of shared ASVs and their total relative abundance within the olive flounder microbiota. **(B)** The number of ASVs present in and shared between the olive flounder and environmental factors. Gray boxes indicate the number of unique ASVs in each group.

We analyzed the microbial composition of environmental factors utilized in aquaculture, including inflowing seawater to the aquaculture facility, seawater from each rearing tank, and commercial feed supplied to the fish. The commercial feed exhibited a relatively simplistic microbial profile, predominantly composed of *Staphylococcus* (40.2%), *Carnimonas* (17.9%), and *Streptococcus* (6.5%) (Supplementary Figure 3). The microbial composition of seawater exhibited high diversity compared to that of olive flounder and commercial feed, with 5.57% of the total seawater microbiota consisting of Archaea. Among the phyla, *Pseudomonadota* consistently showed the highest relative abundance across seawater samples (38.38–45.44%), followed by *Patescibacteria* (12.58–18.91%) and *Bacteroidota* (7.32–19.80%), which also exhibited substantial proportions. To observe changes in the microbial composition of seawater after being introduced into the rearing tank, we compared the microbial composition of seawater before entering the rearing tank and after (Supplementary Figure 4). The taxa that increased in microbial composition in the rearing seawater, compared to the inflowing seawater, included *Flavobacteriia*, *Gammaproteobacteria*, *Gracilibacteria*, and *Verrucomicrobiae* (Supplementary Figure 4). These taxa correspond to core groups constituting the microbial community of the olive flounder. Conversely, the decreased taxa in the rearing seawater comprised *Omnitrophia*, *Alphaproteobacteria*, *Nanoarchaeia*, and *Cyanobacteriia* (Supplementary Figure 4). These taxa are represented by universal components of the microbial community in groundwater or sediments and are involved in nutrient-cycling processes such as photosynthesis. When analyzing the shared ASV between olive flounder and environmental factors, it was found that rearing water shared the most ASVs with olive flounder (Figure 7). Among the 246 ASVs shared with the rearing water, they accounted for 78.5% of the total reads detected in the olive flounder (Figure 7). In contrast, feed shared 56 ASVs with olive flounder, representing only 10.2% of the total reads detected in the flounder. We analyzed the number of ASVs shared between individual mucosal sites of olive flounder and environmental factors to determine the extent of their relationship (Supplementary Figure 5). Among the mucosal sites, the skin shared the largest number of ASVs with Inflow seawater and feed. However, gill showed the highest level of ASV overlap with rearing seawater, shared a total of 183 ASVs (Supplementary Figure 5). In contrast, the intestine and ovary showed lower overall ASV diversity and shared fewer ASVs with environmental factors compared to the external organs (Supplementary Figure 5). Additionally, the microbial composition of feed was not significantly related with any of the mucosal sites, including the intestine and ovary. This pattern of environmental influence on the olive flounder microbiota was consistently observed in the source tracking analysis using the FEAST tool (Supplementary Figure 6). The results show the relative contributions of three sources, which include influent seawater, feed, and unknown origins, to the microbial composition at each mucosal site. These findings further confirm that seawater had a more substantial influence than feed across all sites of the olive flounder.

## Discussion

### Microbial structure and compositional characteristics across mucosal surfaces of olive flounder

The dominant microbial group identified in mature female olive flounders during spawning was the phylum *Pseudomonadota.* Among *Pseudomonadota*, reads belonging to unclassified *Comamonadaceae* and *Vibrio* were the most dominant constituent of olive flounder microbiota (Supplementary Figure 3). However, the microbial structure of each mucosal surface in olive flounder exhibited distinct patterns (Figures 1–4). The four mucosal sites collected for this study—gills, skin, intestines, and ovaries—each reflect distinct regional characteristics in terms of seawater exposure, oxygen concentration, light permeability, and types of host secretions, thereby providing specialized habitats for unique microbiota. We observed that the gills harbored the most diverse species, while the intestines and ovaries exhibited limited species richness. Although the skin is an exposed organ, it exhibited microbial structural similarities to both the gills and the intestines, even including taxa known for their strictly anaerobic nature, such as *Fusobacteria*. Studies on microbial communities in the ovaries of fish have been neglected compared to those on other body sites. In this study, we retrieved qualified DNA for microbial community analysis from ovarian samples of five spawning female olive flounders, whose gonads had not yet entered the degeneration or resorption stages, out of a total of 20 individuals. Consequently, considering the microbial composition, abundance, and beta diversity of the ovarian microbiota, we concluded that its characteristics closely resemble those of the intestinal microbiota, which is also an internal organ. Even though ovarian mucus harbors 79 unique ASVs, it is distinguished from the other three sites (Supplementary Figure 5A).

We examined the distribution of potentially pathogenic taxa within the microbial community of olive flounder. The class *Gammaproteobacteria* includes several genera recognized for their pathogenicity to marine animals, such as *Vibrio* spp., *Shewanella* spp., and *Aliivibrio* spp. (Schrøder et al., 1992; Qin et al., 2014; Ina-Salwany et al., 2019). Family *Brevinemataceae*, *Flavobacteriaceae*, and *Streptococcaceae* also contain species known to cause diseases in fish, including the genera *Brevinema*, *Flavobacterium*, and *Streptococcus* (Weinstein et al., 1997; Nematollahi et al., 2003; Wang et al., 2020). Our analysis confirmed the presence of these potentially pathogenic genera across all mucosal surfaces of the olive flounder. Furthermore, the skin mucosa exclusively contains taxa belonging to *Rickettsiales* and *Chlamydiales*, well known for their obligate intracellular parasitism and ability to cause epitheliocystis in skin and gill of fish (Weisburg et al., 1989; Fryer and Lannan, 1994; Fryer and Lannan, 1996; Stride et al., 2014). Although we identified several opportunistic pathogens across all sites of the olive flounder, these did not lead to actual health issues. While none of the experimental olive flounder used in this study exhibited pathological symptoms, we obtained intriguing results by analyzing the associations between specific microbial compositions and various physiological markers of individual olive flounder, including health, growth, and sexual maturation indicators. The association between microbial community and physiological markers was found most frequently in the skin and least frequently in the gills, reflecting their limited interaction with the host. Among the significant associations identified through multivariable analysis in each site, several key findings stand out. Notably, the abundance of the families *Brevinemataceae* and *Bacillaceae*, in the skin of olive flounder showed a strong positive correlation with blood glucose levels, a stress marker, and also exhibited a positive association with GOT, an indicator of liver damage. Additionally, the abundance of *Brevinemataceae* was significantly associated with higher estradiol levels in both the skin and intestine. In contrast, *Candidatus* Kaiserbacteria and *Gammaproteobacteria*_uc found in the intestine displayed the highest negative correlations with glucose levels, while showing a strong negative association with estradiol (Supplementary Table 6). These findings suggest that controlling specific microbial group in the intestine and skin of olive flounder could be effective in regulating stress induced during the spawning season. Furthermore, the presence of *Terasakiellaceae* in the skin showed a substantial negative correlation with both GOT and GPT levels. *Terasakiellaceae*, comprising the single genus *Terasakiella*, has been isolated from marine and wastewater environments. While its role as a symbiont in fish skin or other hosts remains largely unknown, our findings indicate that this genus warrants further investigation for its potential role in mitigating liver damage in fish hosts.

### Environmental factors influencing microbial composition in olive flounder

Several studies have yielded versatile results regarding the direct influence of the microbial composition of seawater on the mucosal surfaces of fish (Navarrete et al., 2009; Pratte et al., 2018; Guivier et al., 2020; Rosado et al., 2021). In general, gills and skin directly exposed to seawater reflect the microbial composition of seawater more closely, but the gastrointestinal tract is also affected by surrounding waters, especially in early life stages (Navarrete et al., 2009; Wilkes Walburn et al., 2019). In our study, seawater sampled before entering the rearing tank shared 161 ASVs with those found in olive flounder, accounting for 70.1% of the total reads from the olive flounder. After entering the rearing tank, the seawater exhibited a significant structural alteration under the growing influence of the olive flounder, sharing 246 ASVs, which comprised 78.5% of the total relative abundance of olive flounder microbiota (Figure 7). That change was accompanied by a remarkable decrease in the microbial diversity of the seawater. Our observations also revealed that the internal organs of olive flounder exhibited not only lower species richness compared to individual mucosal sites but also weaker associations with environmental factors (Supplementary Figures 2, 5). Furthermore, the intestinal microbiota did not show a strong correlation with the microbial communities of feed, even though feed was considered to have a more direct impact than the relatively inaccessible seawater (Supplementary Figures 5, 6). The microbial composition of commercial feed showed generally low correlations with the microbial communities of olive flounder compared to seawater (Figure 7; Supplementary Figure 6). Considering that commercial feed undergoes sterilization during manufacturing, microbial communities present in the feed are presumed to originate from handling during storage at the fish farm. The detection of *Staphylococcus* in the feed suggests a potential connection to contamination from the handling process by humans. Analysis on the environmental factors revealed that the microbiota of olive flounder is more closely and mutually influenced by seawater than by feed, particularly on the external organs. These findings demonstrate that the external organs of flounder are more susceptible to the influence of seawater microbiota than the internal organs and reaffirm their importance as potential interfaces for regulating host microbiota and facilitating the introduction of specific microbial assemblages.

### Inferring the distinct roles of microbial communities on the mucosal surfaces of olive flounder

The gills, one of the primary excretory organs in fish, play a crucial role in the excretion of ammonia, the most common form of nitrogen waste in aquatic organisms (Wright, 1995; Ip and Chew, 2010). The role of symbiotic microbial communities in the fish gills in ammonia detoxification has been repeatedly highlighted in previous studies (Tarnecki et al., 2016; van Kessel et al., 2016; Legrand et al., 2018; Pratte et al., 2018). Theoretically, ammonia-oxidizing bacteria (AOB) and nitrite-reducing bacteria can neutralize toxic ammonia, thereby contributing positively to fish health (van Kessel et al., 2016). In this study, among the representative taxa performing ammonia-oxidizing functions, *Nitrosomonas* and *Nitrospira* were exclusively found in the gills and skin. Notably, the gills contained significantly higher abundance of *Nitrospira* compared to the other sites (Figure 4). Moreover, the gills showed a significantly higher predicted abundance of nitrite reductase (nirK), one of the core enzymes in the denitrification pathway that produces nitrogen gas through the stepwise reduction of nitrite, compared to other sites. Additionally, both the gills and the skin, as external organs, exhibited higher predicted levels of genes related to denitrification compared to the intestine (Figure 4). Likewise, for sulfur metabolism, genes related to the sulfur oxidizing system were significantly more abundant in the external organs compared to the intestine (Figure 4). Bacteria with sulfur oxidizing functions have the potential to neutralize toxic substances such as thiosulfate, which are commonly present in aquaculture environments, suggesting their beneficial role in maintaining fish health (Petri et al., 2001; Meyer et al., 2007; Krishnani et al., 2010). These findings support previous studies emphasizing the critical role of fish symbiotic microbial communities in detoxification processes and further highlight the distinctive detoxification functions performed by the microbial communities in external mucosal surfaces, particularly the gills. Additionally, another highlighted function predicted through the microbial composition of the gills in olive flounder was the synthesis of SCFAs. SCFAs produced by intestinal microorganisms through the fermentation of carbohydrates are well known to play an important role in maintaining host gut health and epithelial integrity (Macia et al., 2012; Parada Venegas et al., 2019). Phyla such as *Bacillota*, *Bacteroidota*, *Actinomycetota*, and *Verrucomicrobiota* are known as major SCFA producers in the human gut (Fusco et al., 2023). Although, we observed a higher abundance of *Bacteroidota* and *Verrucomicrobiota* in the gills of olive flounder compared to other mucosal sites, the major members of these phyla found in this study lack sufficient evidence supporting SCFA production. Fish gills are generally regarded as an unfavorable condition for the anaerobic fermentation of polysaccharides that leads to SCFA production. Moreover, SCFA synthesis involves complex and diverse pathways across a wide range of bacterial species, and even if the machinery to produce SCFAs is present, its capability can be regulated by the presence of various transporters and enzymes (Mahowald et al., 2009; Tan et al., 2014). Therefore, these predictions do not necessarily indicate actual SCFA production. Whether SCFA synthesis actually occurs in the gill mucus and how it contributes to the overall health of the host requires further investigation.

In addition to the gills, the skin also plays a vital role in host–microbiota interactions and defense mechanisms (Esteban, 2012). Unlike terrestrial animals, the skin of fish is composed of living cells rather than dead keratinized cells and is covered with mucus containing potential nutrients and antimicrobial agents (Gomez and Primm, 2021). The skin of olive flounder did not exhibit distinguishing functional features among the three sites but showed an intermediate pattern between those of the gills and intestines. However, their microbial composition included distinctive taxa from other sites, such as opportunistic pathogens specific to eukaryotic cells, as mentioned above, and bacteria capable of producing antimicrobial substances. In particular, the presence of *Pseudoalteromonas* was highlighted in the skin, a genus well known for producing a wide range of antibiotics capable of inhibiting both prokaryotes and eukaryotes (Bowman, 2007; Offret et al., 2016). Several strains of *Pseudoalteromonas* have been tested for their antagonistic effects against pathogens in marine fish such as European sea bass, clownfish, and yellowtail (Wesseling et al., 2015; Sayes et al., 2016; Rahmani et al., 2023). Conversely, *Pseudoalteromonas* spp. have also been reported to be pathogenic in European sea bass and gilthead seabream (Pujalte et al., 2007). Additionally, *Pseudoalteromonas* has been reported to significantly increase in abundance following antibiotic administration in the skin microbiota of European sea bass and gilthead seabream, suggesting its critical role in the antimicrobial defense of fish skin (Rosado et al., 2019b; Rosado et al., 2023). Our network analysis results on the skin microbial community at the family level revealed a negative correlation between *Pseudoalteromonadaceae* and several families comprising opportunistic bacterial pathogens, such as *Vibrionaceae*, *Shewanellaceae*, *Brevinemataceae* and *Fokiniaceae.* Its negative association with opportunistic pathogens in the skin of olive flounder further supports the potential role of *Pseudoalteromonas* in host antimicrobial defense, warranting further investigation. Our study observed that the skin of olive flounder exhibits intermediate characteristics between the gills and intestines, containing a range of pathogens associated with both internal and external mucosal surfaces. While further research with comparative data across fish species is needed, our findings suggest that the skin microbiota has potential to serve as an indicator of shifts in microbial composition and the presence of pathogens across mucosal surfaces in fish.

Compared to the external mucosal surfaces, the intestinal microbiota displayed distinct metabolic functions and taxonomic profiles. One of the well-known key beneficial effects of the intestinal microbiota is the supply of exogenous vitamins and digestive enzymes that the host cannot synthesize itself. The primary types of vitamins derived from bacterial synthesis include water-soluble B-group vitamins and vitamin K2. The dietary requirement for various vitamins in farmed fish has not been defined in many cases. However, a deficiency of B group vitamins in fish has been reported to lead to decreased appetite and poor growth, while a deficiency of vitamin K can result in increased blood coagulation time and anemia (Kangsen Mai et al., 2022). According to the functional prediction results, the levels of synthetic enzymes for vitamin K2 were predicted to be higher in the intestines. In contrast, the levels for B group vitamins were predicted to be higher in the gills and skin. In the freshwater fish species, *Cetobacterium somerae* is known to serve as a primary supplier of vitamin B12 (Sugita et al., 1991; Tsuchiya et al., 2008). In our study, reads assigned to the genus *Cetobacterium* were frequently found across all sites of the olive flounder, but their average relative abundance was highest in the intestine. Instead, the intestinal microbiota was predicted to have significantly higher Vitamin K2 production levels compared to other sites. Supplying Vitamin K2 from intestinal bacteria could not only benefit fish health but also serve as a cost-effective alternative. This could be an important aspect, as synthetic Vitamin K3, which is commonly used as an additive in aquaculture feed, has an unstable structure that degrades rapidly after being added to feed (Marchetti et al., 1995; Marchetti et al., 1999; Krossøy et al., 2011). In addition to vitamins, another beneficial product that can be synthesized by intestinal microbiota is exogenous digestive enzymes. These enzymes contribute to fish nutrition by enhancing the efficiency of feed utilization. Commercial feeds used in fish farming open contain a high proportion of by-products from poultry or seafood processing, as well as plant-derived materials like soybean meal and wheat bran. These feed components include complex organic carbons such as carbohydrates, cellulose, and chitin, which are difficult for fish to digest directly. It has been shown that adding exogenous digestive enzymes to the diet improves the performance and digestibility of plant-based feeds in Nile tilapia (Lemos and Tacon, 2017; Maas et al., 2020). Likewise, the gut microbiota of fish also serves as a source of enzymes, aiding in the breakdown and digestion of organic matter, which can positively impact fish health (De Schrijver and Ollevier, 2000; Tovar et al., 2002; Mohapatra et al., 2012; Di Maiuta et al., 2013; Llewellyn et al., 2014). In this study, the intestinal microbiota of olive flounder was predicted to have a significantly higher abundance of glycosidases. This result is consistent with previous studies and confirms that the provision of digestive enzymes is a distinct feature of gut symbiotic microbiota, differentiating it from the external organs. In contrast to the beneficial roles of the intestinal microbial community highlighted earlier, functions related to LPS synthesis and antibiotic resistance are notable as they represent potential mechanisms that could negatively impact the host. Lipopolysaccharide (LPS) derived from the lipid outer membrane of bacteria is a well-known inducer of inflammatory responses. In humans, LPS is considered a major virulent factor and a marker of dysbiosis in gut Gram-negative bacteria. However, in fish, which exhibit resistance to endotoxic shock, LPS is regarded as a potent stimulator of the innate immune system (Swain et al., 2008; CHEN et al., 2010; Wang et al., 2019). The synthesis of LPS, which is predicted to be particularly abundant in the intestine, could have controversial implications for the host, requiring further investigation. Another noteworthy finding from intestinal microbiota is the significantly higher abundance of resistance genes against beta-lactam and phenicol antibiotics compared to other sites. While KOs related to beta-lactam antibiotic biosynthesis were significantly enriched in the gills. These findings indicate that the origin of antibiotic resistance genes in the intestine is not associated with antibiotic-producing bacteria. Furthermore, Streptomyces, a well-known producer of various kinds of antibiotics, including chloramphenicol, was not detected in any of the mucosal sites of olive flounder. Among the bacteria associated with beta-lactam antibiotic production, *Flavobacterium*, and *Pseudomonas* were identified in relatively small quantities. *Flavobacterium* was present in low abundance in the gills and skin, while *Pseudomonas* was rarely detected but appeared in trace amounts in the skin and intestines of a few individuals. The most abundant phenicol resistance gene identified in the intestine was K00638 (chloramphenicol O-acetyltransferase type B), the same type found on the chromosome of *Vibrio cholerae*. Similarly, the most prevalent beta-lactamase in the intestine was K19217 (beta-lactamase class A CARB-17), corresponding to the type reported in *Vibrio parahaemolyticus*. These functional prediction results suggest that the antibiotic resistance genes in the intestine are likely influenced by the abundance of *Vibrio* species within the intestinal microbiota. The results emphasize the importance of the intestinal microbiota as a driving force behind both beneficial metabolic processes and potential health risks in olive flounder. Through their composition, these analytical findings provide a deeper understanding of the complex functions of microbial communities, but we also underscore the need for validation through experimental evidence.

## Conclusion

This study comprehensively analyzes the microbial communities across various mucosal surfaces of olive flounder, highlighting their distinct structural and functional characteristics. The external mucosal sites, such as the gills and skin, were characterized by unique metabolic pathways, including photosynthesis and sulfur-oxidizing systems. These functions may play a crucial role in adapting to direct exposure to seawater, potentially enhancing mucosal barrier protection and nutrient acquisition. In contrast, the internal organ, represented by the intestine, exhibited a simpler microbial composition dominated by *Gammaproteobacteria* and displayed characteristic functional properties, including polysaccharide degradation, LPS synthesis, and vitamin K2 production. The study also identified the presence of potentially pathogenic taxa, such as *Vibrio* spp., *Brevinema*, *Rickettsiales*, and *Chlamydiales*, across multiple mucosal surfaces. We have highlighted microbial groups that may potentially influence host immunity by identifying the distribution patterns of opportunistic pathogens and their associated taxa across different mucosal sites. Additionally, an analysis of the microbial composition of environmental factors suggests that managing the microbial communities in rearing seawater could be an effective strategy for modulating the mucosal microbiota of olive flounder. Analyzing physiological markers, we identified microbial taxa correlated with stress and health indicators, highlighting potential candidates that may positively impact fish health. Although this study provides valuable insights into the ecological roles of mucosal microbiota in olive flounder, the findings are based on predicted functions using an amplicon sequencing approach. Future studies should employ metagenomic or transcriptomic approaches to validate the actual microbial functions. Furthermore, as this study focused on a specific life stage (spawning period) and environmental conditions, additional research is needed to fully characterize the intrinsic microbiota of olive flounder across diverse developmental stages and habitats. Future studies should also aim to validate these findings by examining probiotic strains and exploring intervention strategies for developing microbiota-based solutions to enhance olive flounder health and improve the sustainability of aquaculture practices.

## Data Availability

The raw reads data obtained from olive flounder and environmental samples in this study have been deposited in the NCBI Sequence Read Archive (SRA) database under accession number PRJNA1229132.
