# Supplementary material for: Microbial community structure and functional characteristics across the mucosal surfaces of olive flounder (Paralichthys olivaceus)
Source: Front Microbiol. 2025 May 21;16:1587288. doi: 10.3389/fmicb.2025.1587288 (PMC12134082; doi:10.3389/fmicb.2025.1587288)
Supplement: Supplementary file 2 [file Data_Sheet_1.docx]

Supplementary Material

# Supplementary Figures


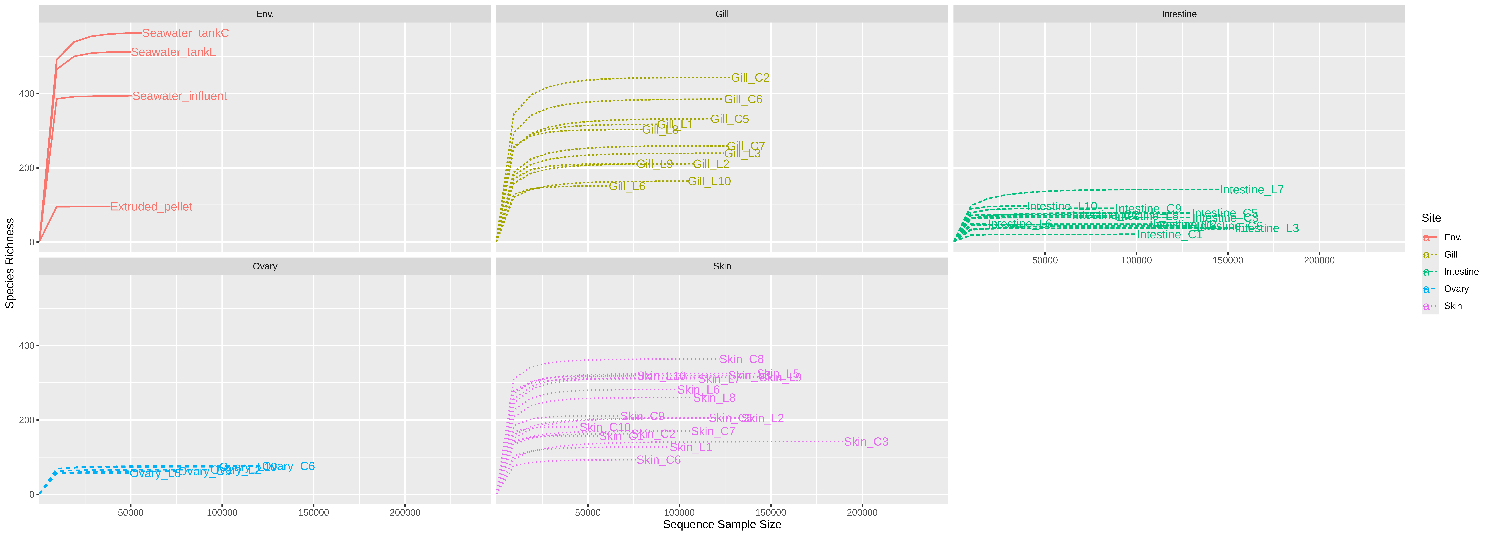


Supplementary Figure 1. Rarefaction curves of microbial communities obtained from four mucosal sites of olive flounder (gill, intestine, skin, ovary) and from environmental samples (seawater and feed).


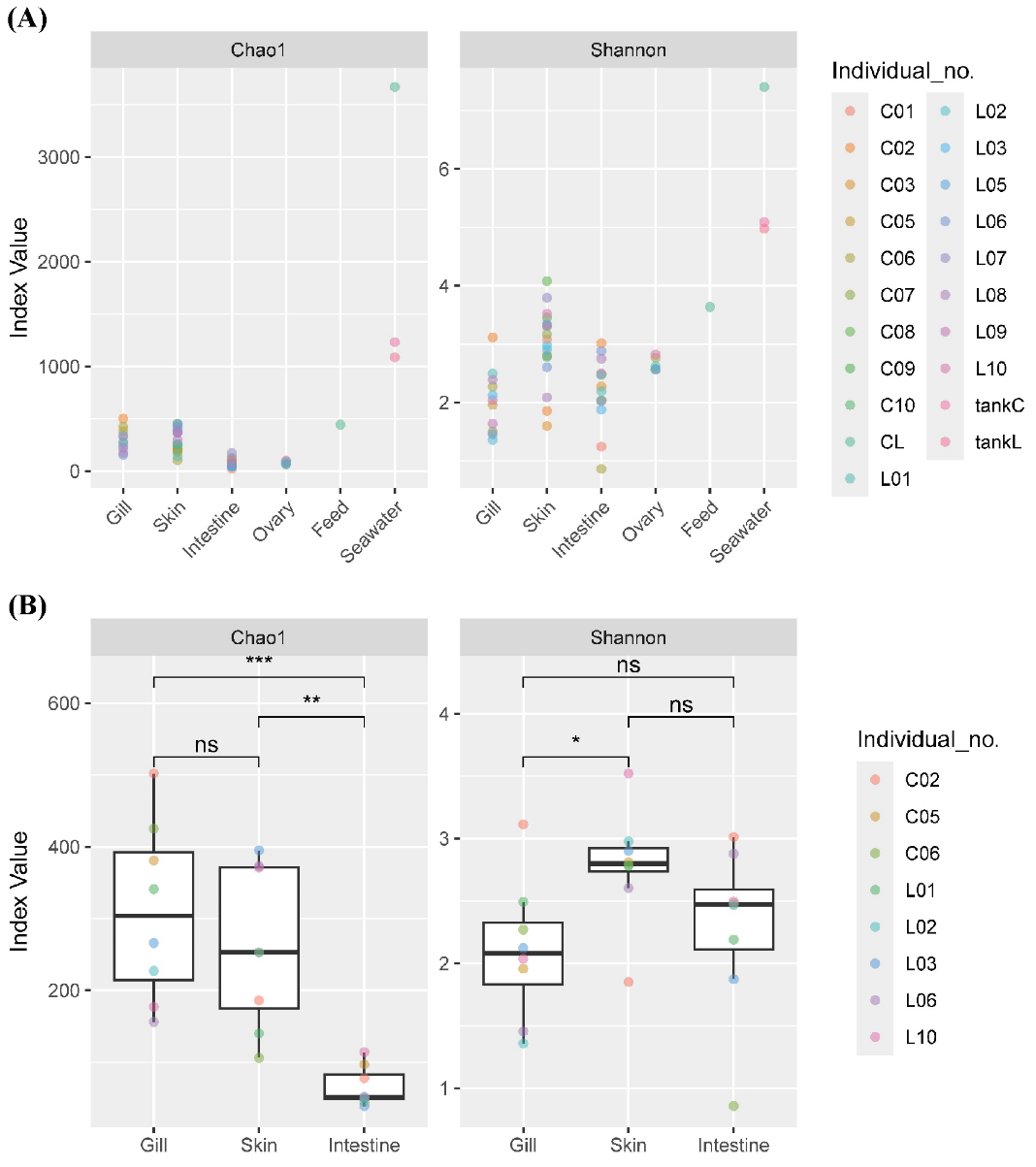


Supplementary Figure 2. Alpha Diversity Summary. The richness and diversity indices are represented by Chao1 and Shannon indices, respectively. Color of each points indicates individual number of fish from two culture tank (C/L). (A) Diversity indices of entire sample by group. (B) Diversity indices of the R8 group (comprising eight fish: C2, 5, 6, and L1, 2, 3, 6, 10). Comparisons between the two groups were performed using the Wilcoxon test. Significance levels are indicated as follows: "***" for p-values between 0.0001 and 0.001, "**" for p-values between 0.001 and 0.01, "*" for p-values between 0.01 and 0.05, and "ns" for p-values between 0.05 and 1 (not significant).


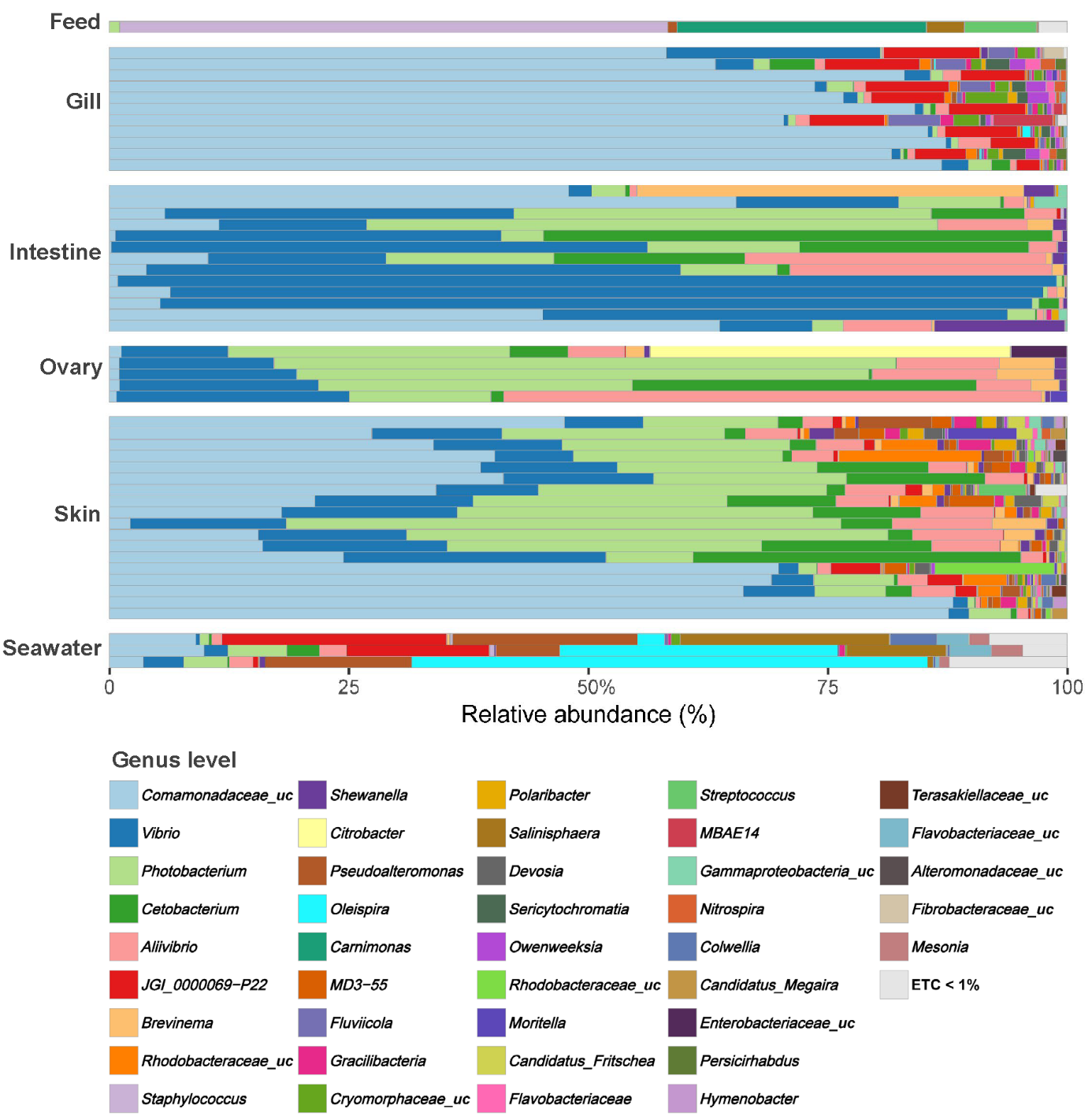


Supplementary Figure 3. Microbial composition at the genus level across all samples, including olive flounder, feed, and seawater. The top 41 genera with a relative abundance greater than 1% in at least one sample are presented.


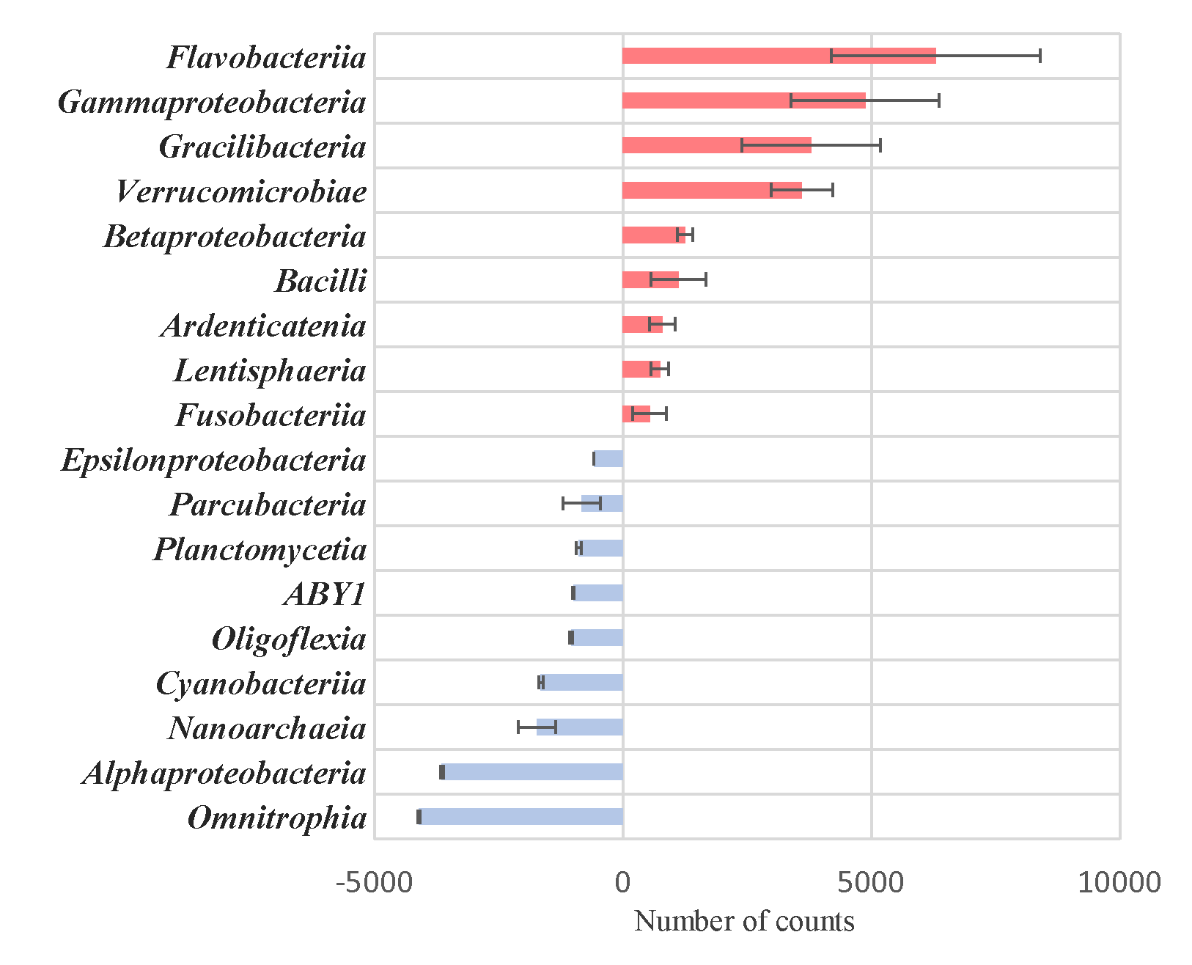


Supplementary Figure 4. Fluctuation bar chart showing microbial composition changes in seawater after introduction into rearing tanks. Only the 18 classes with the largest fluctuations are displayed. Bars represent the average change between influent seawater and both rearing tanks, while error bars indicate the variation in changes between the two rearing tanks.


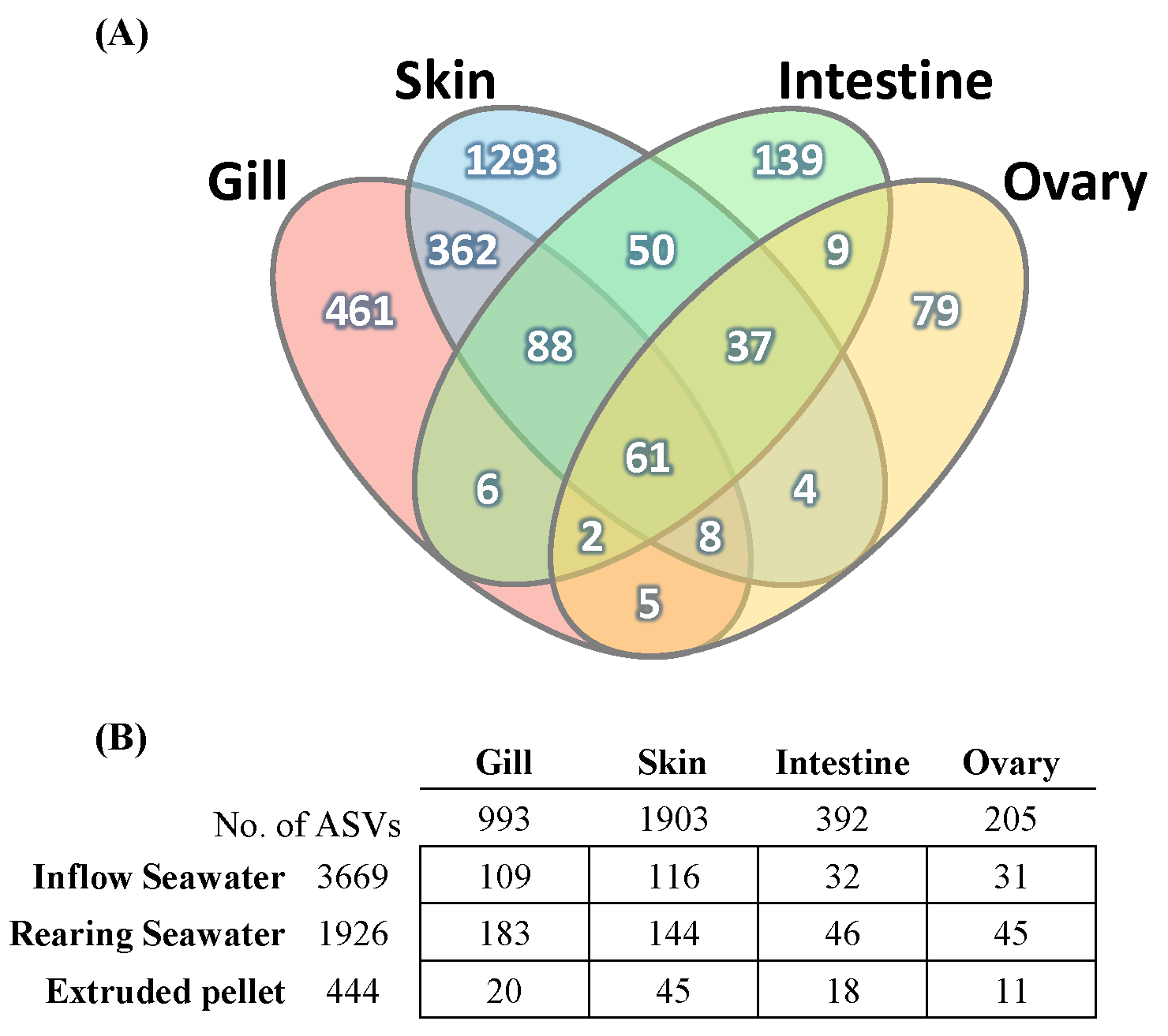


Supplementary Figure 5. Relationship between each mucosal surface of olive flounder and environmental factors. **(A)** Venn Diagram of ASVs shared by four mucosal surfaces. **(B)** The number of ASVs present in and shared between four mucosal surfaces of olive flounder and environmental factors.


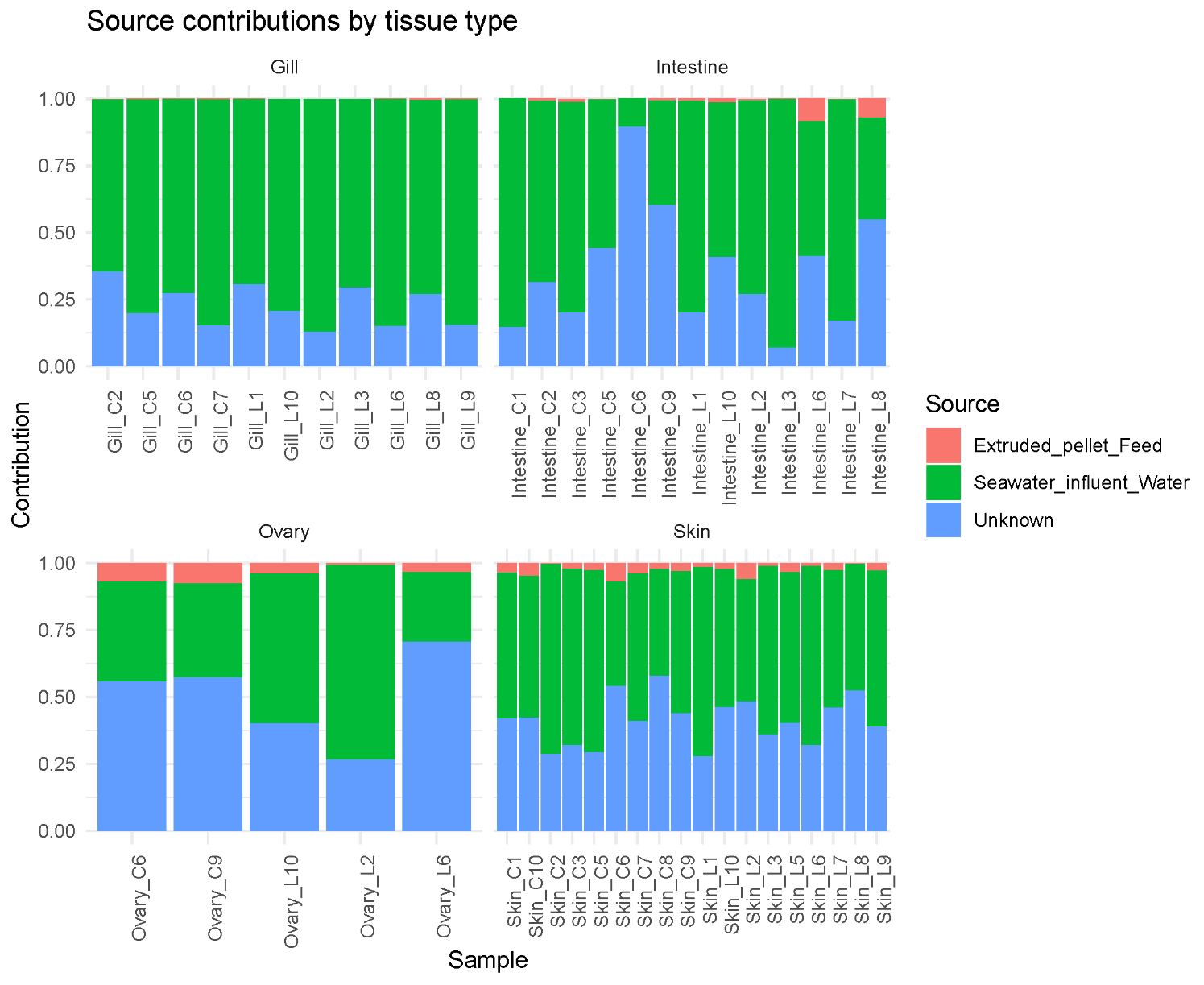


Supplementary Figure 6. FEAST-based source tracking of microbiota from four mucosal tissues of olive flounder. Stacked bar plots show the estimated contributions of environmental sources to the microbiota of gill, intestine, ovary, and skin samples.
